# Saffron Reduced Toxic Effects of its Constituent, Safranal, in Acute and Subacute Toxicities in Rats

**DOI:** 10.17795/jjnpp-13168

**Published:** 2014-02-20

**Authors:** Toktam Ziaee, Bibi Marjan Razavi, Hossein Hosseinzadeh

**Affiliations:** 1 Department of Pharmacodynamics and Toxicology, School of Pharmacy, Mashhad University of Medical Sciences, Mashhad, IR Iran; 2 Targeted Drug Delivery Research Center, Department of Pharmacodynamy and Toxicology, School of Pharmacy, Mashhad University of Medical Sciences, Mashhad, IR Iran; 3 Pharmaceutical Research Center, Department of Pharmacodynamy and Toxicology, School of Pharmacy, Mashhad University of Medical Sciences, Mashhad, IR Iran

**Keywords:** Crocus, Toxicity Tests, Sub-acute, Medicine, Traditional

## Abstract

**Background:**

Saffron and its constituents are widely used around the world as a spice and medicinal plant. Different constituents in medicinal herbs are thought to have the potential to induce useful and/or adverse effects. So, efforts have been made to find the best and most valuable tools to reduce their adverse effects.

**Objectives:**

According to Iranian traditional medicine (ITM), it is believed that administration of whole herbs exhibits more activity and fewer side effects than isolated constituents. Since toxicological studies have indicated that safranal is more toxic than other active components in saffron stigma, thus this study was undertaken to evaluate the effect of co-administration of saffron extract and safranal in acute and sub-acute toxicities in rats.

**Materials and Methods:**

In acute toxicity, rats received safranal (1.2 mL/kg, IP) plus saffron aqueous extract (25-100 mg/kg, IP). One and four days after the treatment, percentage of mortality was assessed. In subacute toxicity, rats were randomly divided into six groups. Group 1) safranal (0.2 mL/kg, IP), Groups 2, 3 and 4) safranal plus saffron aqueous extract (5, 10 and 20 mg/kg, IP) Groups 5 and 6) Paraffin and normal saline, as solvents of safranal and saffron aqueous extract, respectively. Treatments were continued for 21 days. For sub-acute toxicity, the percentages of lethality as well as some biochemical parameters were evaluated.

**Results:**

Our results showed that four days co-treatment of safranal and saffron significantly reduced mortality, so that the effect was more obvious in lower doses. Sub-acute toxicity studies showed that saffron could increase survival in rats so that no mortality was observed at dose of 10 mg/kg. Our data also indicated that the levels of triglyceride, BUN and ALT significantly increased after sub-acute interaperitoneal (IP) administration of safranal (0.2 mL/kg/day) and co-treatment of saffron aqueous extract (5 and 10 mg/kg) plus safranal significantly improved all toxic effects of safranal on biochemical parameters.

**Conclusions:**

The co-administration of saffron aqueous extract and safranal reduced toxic effects of safranal in acute and sub-acute toxicities.

## 1. Background

Nowadays, there has been increasing interest in the use of herbal medicines and natural products for the treatment of a variety of disorders (1). *Crocus sativus*, known as saffron, is a member of the Iridaceae family. It has been extensively used as an aphrodisiac, antispasmodic and expectorant in folk medicine (2). Furthermore various pharmacological studies have been demonstrated that saffron stigma extracts exhibit different beneficial properties, including anticonvulsant (3), antidepressant (4), antinociceptive, anti-inflammatory (5), antioxidant (6), antitussive (7), improving memory and learning ability after chronic cerebral hypoperfusion (8) hypotensive effects (9). Saffron and its active components also showed protective effects on diazinon and acrylamide induced oxidative stress (10-13). Saffron has been used medicinally in many countries all over the world. Recently, there has been rising trends towards the adverse effects of herbal medicine (14). A few reports have been published regarding the toxic effects of saffron. Toxicological studies showed that LD50 values of saffron stigma and petal extracts by intra-peritoneal administration in mice were 1.6 and 6 g/kg, respectively. Sub-acute toxicity studies, showed that saffron stigma and petal extracts decreased the value of hematocrit, hemoglobin and erythrocytes, however, the stigma extract did not cause any significant pathological effects in different organs (15).

Safranal, the main component of *C. sativus* essential oil is thought to be responsible for the unique odor of saffron (16). In addition to the effectiveness of safranal for a variety of disorders including depression (4), anxiety (17), convulsion (18), nociception and inflammation (19), studies have shown that safranal could exhibit toxicity. Previous studies showed that a significant reduction in body weight, hematological and biochemical parameters was evident following safranal administration (0.1, 0.25 or 0.5 mL/kg/day, orally) after 21 days (20). Furthermore toxic effect of safranal, in some tissues (kidneys and lung) especially at the dose of 0.5 mL/Kg was observed. According to studies on toxicity of major components of saffron, it seems that safranal is more toxic than the others. With regards to the widespread use of herbal products because of their efficacy and cultural acceptability, efforts have been made to find the best and most valuable tools to reduce their adverse effects (21). In Iranian Traditional Medicine (ITM), there have been reports on the uses of whole plant to reduce some adverse effects induced by plants containing toxic ingredients. For example, it has been recommended that the consumption of *Prunus armeniaca* whole fruit could reduce gastric inflammation induced by *P. armeniaca* seeds (22).

## 2. Objectives

The present study was undertaken to evaluate the hypothesis that co-administration of saffron aqueous extract, as a safe preparation, could diminish some adverse effects induced by safranal in acute and sub-acute toxicities.

## 3. Materials and Methods

### 3.1. Chemicals and Plants

The aqueous extract of *C. sativus* was prepared by the maceration method. Briefly, 8 g of stigma powder was macerated in 300 mL of distilled water for 72 hours with continuous shaking in the refrigerator. The supernatant was separated by centrifugation and transferred to a Freeze-drier. After 24 hours, lyophilized powder of the extract was available. Safranal was purchased from Fluka Chemie AG (Buchs, Switzerland). Enzymatic reagent kits for determination of alanine aminotransferase (ALT), aspartate aminotransferase (AST) and lactic acid dehydrogenase (LDH) were purchased from Greiner Bio-One and bilirubin, blood urea nitrogen (BUN), albumin, cholesterol and triglyceride were purchased from Pars Azmon Co. All other chemicals and solvents used throughout this study were of analytical grade.

### 3.2. Animals

Male Wistar rats (weighing approximately 200-250 g) were obtained from the animal house of the Pharmacy School of Mashhad University of Medical Sciences. Animals were housed in a colony room under a 12 hour light/dark cycle at 21 ± 2˚C and had free access to water and food. All animal experiments were approved by the Animal Care Committee of the Mashhad University of Medical Sciences.

### 3.3. Acute Toxicity

Animals were randomly divided into several groups (n = 6). The first group (control group) received paraffin and other groups were treated with different doses of safranal via intraperitoneal (IP) injections. Following administration, animals were observed for signs of toxicity and mortality for a period of 24 hours after treatment. The lethal dose (LD50) was estimated according to the method described by Litchfield and Wilcoxon (PHARM/PCS software version 4). In another experiment, rats received safranal (1.2 mL/kg, IP) plus saffron aqueous extract (25-100 mg/kg, IP). One and four days after treatments, the percentages of mortality were assessed.

### 3.4. Subacute Toxicity

Rats were randomly divided into six groups. Group 1) safranal (0.2 mL/kg, IP), Groups 2, 3 and 4) safranal plus saffron aqueous extract (5, 10 and 20 mg/kg, IP) Groups 5 and 6) Paraffin and normal saline, as solvents of safranal and saffron aqueous extract, respectively. Treatments were continued for 21 days. The body weight was determined weekly. Animals were observed for general behavioral and signs of abnormalities during the experiment duration.

### 3.5. Blood Sampling

After 21 days, animals were anaesthetized by chloroform. Blood samples were collected by cardiac puncture into sterile tubes for biochemical tests. Blood samples were centrifuged at 5000 rpm for 15 minutes and serum was separated.

### 3.6. Biochemical Analysis

The levels of AST, ALT, LDH, total bilirubin, serum glucose, total cholesterol, triglyceride, albumin, BUN and creatinine were determined using commercial colorimetric kits.

### 3.7. Statistical Analysis

Data were determined as Mean ± SEM. All data were analyzed using analysis of variance (ANOVA) followed by Tukey- Kramer. Statistical significance was defined as P values less than 0.05 (P < 0.05).

## 4. Results

### 4.1. Acute Toxicity Studies

**Figure 1.**
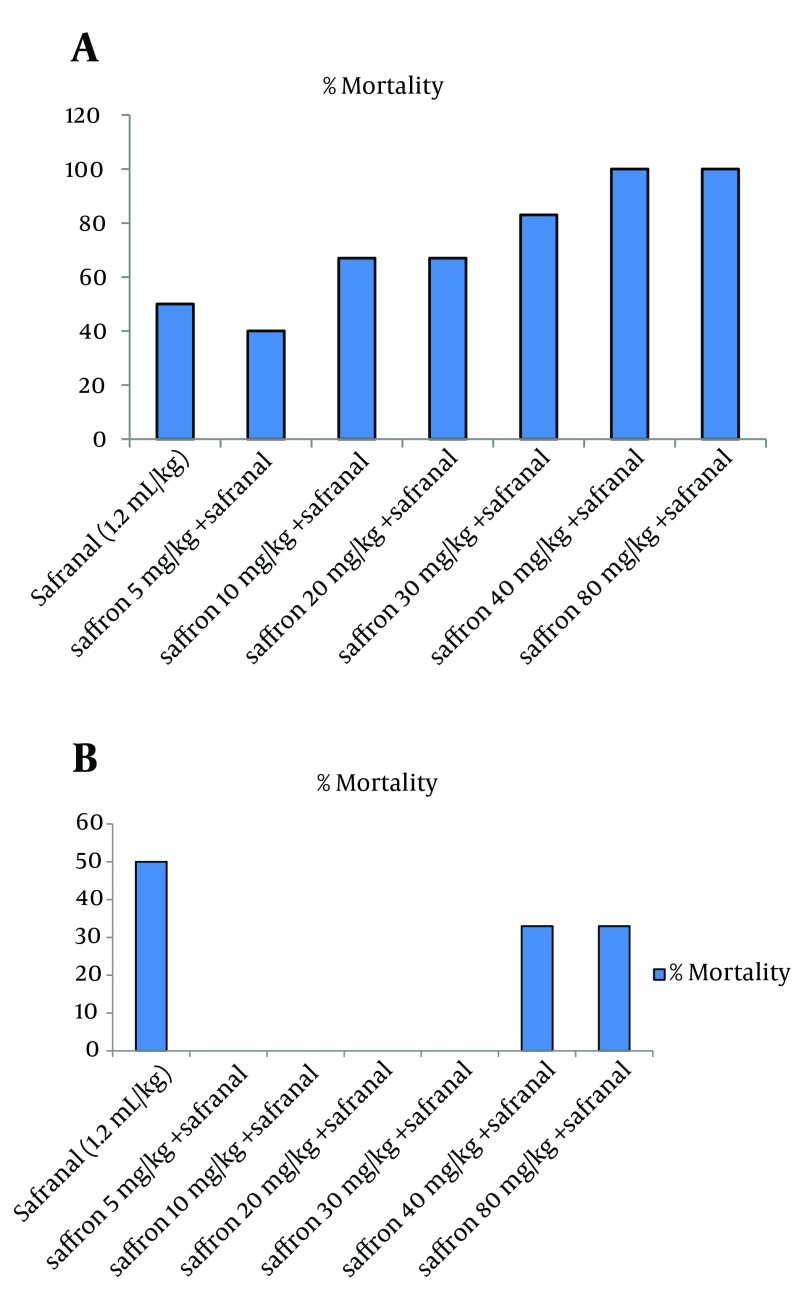
Effect of Saffron Aqueous Extract on Safranal Acute Toxicity Rats treated for one A. and four B. consecutive days. Data shows mortality, % (n =6).

The LD50 values of safranal in male rat were calculated as 1.2 mL/kg via IP injection. The concurrent administration of safranal (1.2 mL/kg) and saffron aqueous extract (5, 10, 20 and 30 mg/kg) after four days caused a significant reduction in percentage of mortality in comparison with safranal (P < 0.001) (Figure 1B). The results also showed that lethality increased with increasing of saffron doses (40 and 50 mg/kg). One-day co-administration of safranal and saffron did not reduce mortality as compared to safranal (Figure 1A). 

### 4.2. Subacute Toxicity Studies

**Figure 2.**
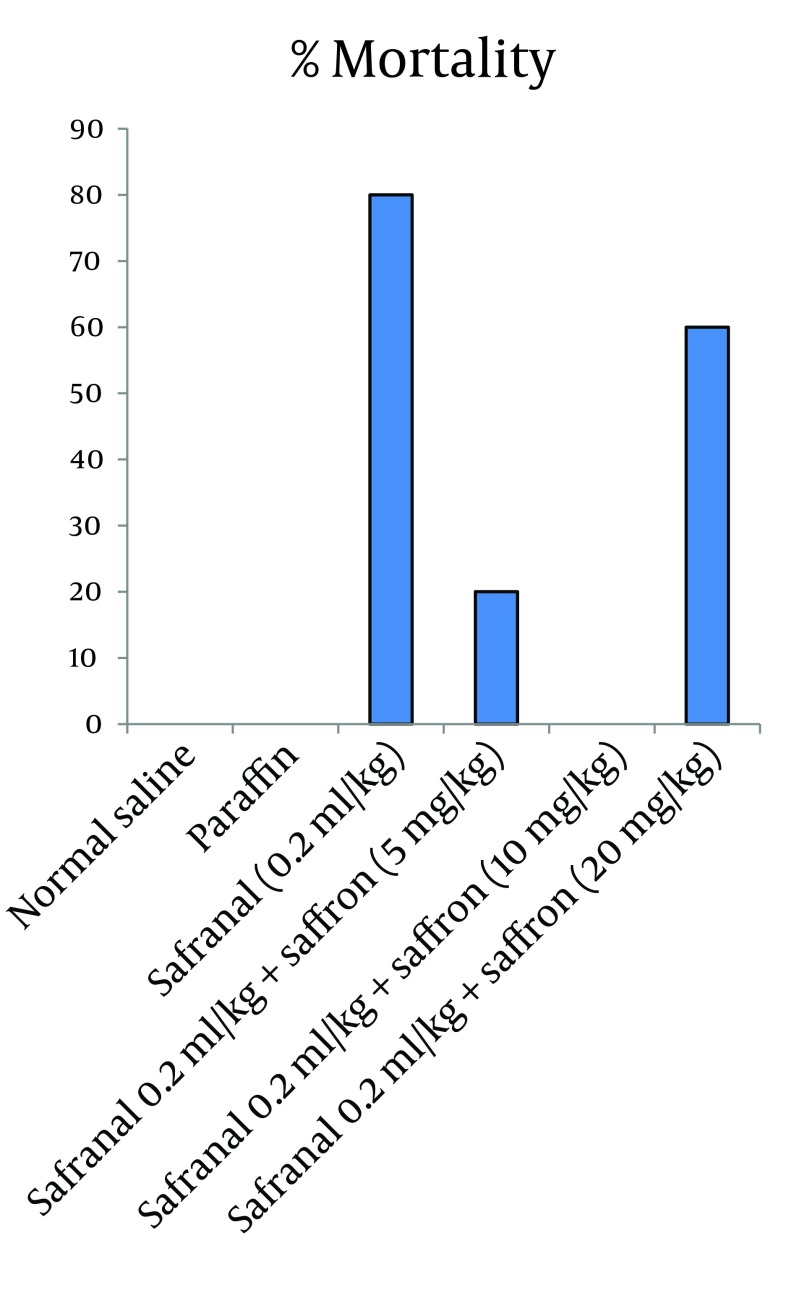
Effect of Saffron Aqueous Extract on Safranal Sub Acute Toxicity Rats treated for 21 days. Data shows mortality, % (n = 6).

**Figure 3.**
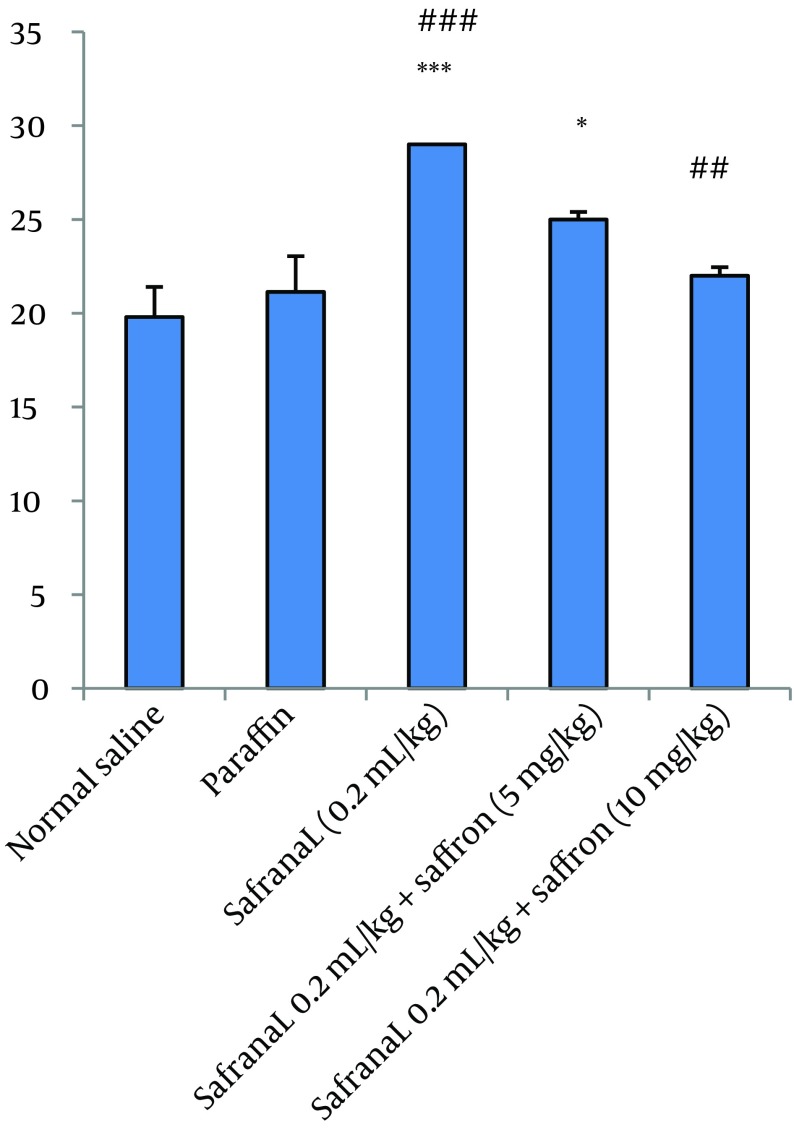
Effects of Saffron Aqueous Extract and Safranal on A. BUN, B. ALT, and C. TG. Rats treated for 21 days. Data shows Mean ± SEM (n = 6) * P < 0.05 and *** P < 0.001 vs control, #P < 0.05, ## P < 0.01 and ### P < 0.001 vs safranal group.

In this model of experiment, safranal (0.2 mL/kg/day) plus saffron aqueous extract (5, 10 and 20 mg/kg/day) were administrated IP for 21 days. The results showed that saffron aqueous extract significantly decreased mortality in comparison to safranal. The effect of saffron aqueous extract at 10 mg/kg was more than other doses. Increasing dose of saffron to 20 mg/kg caused an increase in mortality (Figure 2). The results of biochemical evaluation also showed that sub-acute IP administration of safranal (0.2 mL/kg/day) did not show any significant difference in some biochemical profiles such as total cholesterol, serum glucose, serum creatinine, bilirubin, AST and LDH (Table 1). However, the levels of triglyceride, BUN and ALT showed a significant increase in safranal and co treatment of saffron aqueous extract (5 and 10 mg/kg) plus safranal significantly improved all toxic effects of safranal on biochemical parameters (Figure 3). 

**Table 1.** Effects of *Saffron aqueous *Extract and Safranal on Serum Biochemical Parameters in Rats Treated for 21 Consecutive Days ^a,b ^

Parameter	Control	Safranal 0.2, mL/kg	Safranal + Saffron 5, mg/kg	Safranal + Saffron 10, mg/kg
**Glucose, mg/dL**	231.60 ± 28.90	234.00	248.00 ± 9.40	214.00 ± 2.30
**Creatinine, mg/dL**	0.31 ± 0.01	0.30	0.30	0.30
**Cholesterol, mg/dL**	100.60 ± 10.7	93.00	110.70 ± 1.50	101.7 ± 2.9
**LDH, IU/L**	211.00 ± 20.20	170.30	258.00	164.20 ± 27.30
**AST, IU/L**	290.00 ± 24.20	245.00	275.00 ± 6.07	291.00 ± 33.70
**Total bilirubin, mg/dL**	0.10 ± 0.03	0.10 ± 0.01	0.10 ± 0.01	0.10 ± 0.01

^a^ Abbrevitions: AST, aspartate aminotransferase; LDH, lactic acid dehydrogenase.

^b^ Data showed as Mean ± SEM and (n = 6).

## 5. Discussion

Saffron and its constituents are widely used around the world as a spice and medicinal plant (2). Different constituents in medicinal herbs are thought to have the potential to induce useful and/or adverse effects (23). In Iranian traditional medicine (ITM), it is believed that consumption of whole herbs exhibit more activity and less side effects than isolated constituents (22). Since toxicological studies indicated that safranal is more toxic than other active components in saffron stigma in pharmacological ranges (20, 24, 25), this study was undertaken to evaluate the effect of co-administration of saffron extract and safranal in acute and sub-acute toxicities in rats. Our results showed that four days co-treatment of safranal and saffron significantly reduced mortality, so that the effect was more obvious in lower doses. It seems that the increased amount of harmful constituents in high doses of saffron caused partially more adverse effects rather than lower doses. The co-treatment of saffron and safranal did not decrease lethality induced by safranal after one day. It could be concluded that one-day co-treatment is not sufficient to reduce safranal toxicity by saffron. It seems that adequate time is required for saffron to modulate disorders induced by safranal. Sub-acute toxicity studies showed that saffron could increase survival in rats so that no mortality was observed at dose of 10 mg/kg. Similar to the results of acute toxicity, increasing dose of saffron caused an increase in mortality. This also could be partially related to the increased level of toxic substances in high doses. Our data also indicated that sub-acute IP administration of safranal (0.2 mL/kg/day) does not show any significant difference in some biochemical parameters such as total cholesterol, serum glucose, serum creatinine, LDH, AST and bilirubin. However, the levels of triglyceride, BUN and ALT showed a significant increase in safranal and co-treatment of saffron aqueous extract (5 and 10 mg/kg), plus safranal significantly improved all toxic effects of safranal on biochemical parameters.

Iranian Traditional Medicine (ITM) has shown that by the usage of certain standardized constituents instead of whole herbs, valuable efficacy of whole herbs will be missed (22). The reason is probably that whole herbs work best as body modulator and transformers, especially facilitating the immune system, providing the foundation of the body to begin healing itself (26). Saffron stigma consists of more than 150 chemicals (27), some of them possess valuable and beneficial properties and some exhibit undesirable and harmful activities. The consumption of saffron stigma instead of its main components alone, might increase tolerance and modulate abnormalities in the body. In folk medicine, it has been reported that consumption of whole plants or fruits could reduce some adverse effects induced by some constituents, for example, it has been recommended that consumption of Prunus armeniaca whole fruit, could reduce gastric inflammation induced by the P. armeniaca seed (22). Previous studies have shown that six weeks administration of safranal (0.25 and 0.5 mL/kg/day, orally) in diabetic rats reduces fasting blood glucose and HbA1c levels and improves the blood insulin levels significantly, there were no significant changes in the blood SGOT, SGPT and creatinine levels (28). Moreover the results of our previous study revealed that safranal (0.1, 0.25 or 0.5 mL/kg/day, orally) causes sedation, relaxation and reduction in locomotor activity. Decrease in food and water consumption and weight loss were also observed. A significant reduction in some hematological parameters was also demonstrated. Furthermore safranal reduced total cholesterol and triglyceride at all doses and ALP at two higher doses. In addition, safranal (0.5 mL/kg) caused an increase in the levels of LDH and BUN. Histopathological examination indicated that safranal, especially at the dose of 0.5 mL/Kg caused toxicity in kidneys and lungs (20). In this study, safranal (0.2 mL/kg) increased the levels of some common markers of liver toxicity such as ALT (SGPT). However, the levels of bilirubin, AST (SGOT) and LDH were not affected by safranal. It may be postulated that safranal causes mild to moderate hepatotoxicity. Saffron plus safranal especially at dose of 10 mg/kg reduced toxicity. The hepato-protective effects of saffron and its active component, crocin, have been shown previously (29). Crocin improved toxic effects of diazinon on rat livers through its antioxidative properties. It could be suggested that there are some active components in saffron stigma aqueous extract which have high antioxidant effects and due to the presence of these chemicals, saffron could improve safranal toxic effects on liver, but increasing the dose may decrease beneficial effects. Our results also indicated that safranal (0.2 mL/kg) increased BUN as a predictor of renal insufficiently while the amount of creatinine was unchanged. Renal damage induced by safranal was established previously (20). Similar to our results, safranal treated rats showed an increase in BUN and no difference in the levels of serum creatinine was observed. Also these observations were confirmed by renal histopathologic damages. Our results also showed that saffron stigma could decrease the elevation of BUN induced by safranal. The protective effects of saffron extracts against renal toxicity have also been reported in some studies (30, 31).

The results of safranal on lipid profile showed that although TG was increased, the level of total cholesterol was unchanged. The results of a previous study showed that both TG and cholesterol decreased following safranal administration (20). The inconsistency between this study and our previous research might be due to the differences in route of administration and/or the differences in the safranal doses. The alterations in lipid profile induced by safranal improved with co-administration of saffron. In summary, our results showed that co-administration of saffron aqueous extract and safranal reduces toxic effects of safranal in acute and sub-acute toxicity as evident by the reduction in mortality as well as alleviation of some safranal toxic effects on specific biochemical markers. Furthermore based on the results, it could be recommended that the consumption of saffron as a whole plant exhibits more safety than safranal as an active component.
